# UzbekPOS: A multi-domain dataset for Uzbek part-of-speech tagging

**DOI:** 10.1016/j.dib.2026.112640

**Published:** 2026-02-28

**Authors:** Maksud Sharipov, Elmurod Kuriyozov, Jernej Vičič

**Affiliations:** a Urgench State University named after Abu Rayhan Biruni, 14, Kh. Alimdjan str, Urgench 220100, Uzbekistan; b University of Primorska, FAMNIT, Glagoljaska 8, Koper 6000, Slovenia

**Keywords:** POS tagging, Uzbek language, Morphological annotation, Natural language processing

## Abstract

In this paper, we introduce **UzbekPOS** — a part-of-speech (POS) tagged dataset manually annotated for the Uzbek language, designed for natural language processing, artificial intelligence models, and corpus linguistics applications. This tagged corpus is currently the **largest publicly available POS-tagged corpus for the Uzbek language**. The dataset comprises sentences drawn from a diverse range of Uzbek text sources, including literature, news outlets, science, education, and public speaking, to reflect linguistic and topical diversity. The sentences are tokenized and annotated by professional annotators, utilizing a finely grained POS tagset which integrates standard Universal Dependencies with additional labels that are specific to the morphological and syntactic features of the Uzbek language, comprising 16 tags in total.

The UzbekPOS contains almost 4.5K sentences and more than 53K token/tag pairs, with each annotation cross-verified by at least two annotators for highest reliability. It also comes with both raw (txt) and generally accepted formats of distribution (TSV, JSON), as well as the universal POS-tagging format (conllu). This resource is one of the first and the largest openly published POS-tagged dataset for Uzbek, an under-resourced and morphologically complex Turkic language. This dataset can also act as a key foundation for training POS taggers, as a test set for machine learning models, and as a source for linguistic studies. The resource also bears the reusability potential for tasks of related kinds, such as morphological analysis, syntactic parsing, and transfer learning across languages of the Turkic family. Furthermore, this dataset can serve as seed material for creating similar corpora of POS for other Turkic languages and can help conduct cross-linguistic analyses and tool building.

Specifications Table

**Table:** 

Subject	Computer Science, Linguistics;
Specific subject area	Natural language processing, Computational Linguistics, Corpus Linguistics;
Type of data	Text corpus (sentences, tokens); Json, TSV, Conllu formatted data.
Data collection	Raw text sentences were gathered from various sources of Uzbek texts, such as literary texts, news articles, and teaching materials. Texts are balanced over fields, tokenized and normalized before annotation. Manual POS tagging was done by several expert annotators using a pre-defined tagset, while disagreement among them was resolved by adjudication.
Data source location	Higher educational institution: Urgench State University named after Abu Rayhan Biruni 14, Kh.Alimdjan str, Urgench city, 220100, Uzbekistan
Data accessibility	1. Repository name: Mendeley data - UzbekPOS: Multi-domain Part-Of-Speech Dataset for the Uzbek Language Data identification number: 10.17632/55f889ncnx.1 Direct URL to data: https://doi.org/10.17632/55f889ncnx.1 2. Repository name: Github - UzbekPOS: Multi-domain Part-Of-Speech Dataset for the Uzbek Language Direct URL to data: https://github.com/MaksudSharipov/UzbekPOS 3. Code availability: The Python scripts used to generate the corpus statistics and tag distributions (Table 3, Table 4) are available in the GitHub repository.
Related research article	The older version of the dataset was used in this research paper: *M. Sharipov, E. Kuriyozov, O. Yuldashev, and O. Sobirov, ‘UzbekTagger: The rule-based POS tagger for Uzbek language’, in 10th Language & Technology Conference: Human Language Technologies as a Challenge for Computer Science and Linguistics, 2023.*

## 1 Value of the Data

• The largest publicly available POS-Tagged dataset for the Uzbek language. These annotated sentences present an openly available expert-annotated corpus of POS for Uzbek that is the largest in size compared to the ones publicly available. This resource allows for further and more intrinsic experiments in the natural processing of the language.
• Supports Natural Language Processing (NLP) tool construction and benchmarking. The presented dataset is reusable in terms of training and testing POS taggers, morphological analyzers, and syntactic parsers. Predefined train, development, and test sets are included in the dataset repository to allow standardized benchmarking and comparison of created models.
• Supports cross-linguistic and comparative research. Given that the annotation guideline followed in this dataset follows the format of the Universal Dependencies [1] with little additions for Uzbek-specific tags and rules, it is perfect for cross-linguistic and typological analyses of Turkic languages. These features can be used for a comparative basis of low-resource highly inflectional languages scenarios.
• Valid resource for both educational and applied settings. The created dataset serves in the teaching of corpus linguistics, NLP, and computational linguistics classes and in the creation of Uzbek-language applications, such as intelligent tutoring systems, spell and grammar checkers, to name a few.
• Framework for transfer to other Turkic languages. Apart from Uzbek, the corpus serves as a blueprint to create parallel sets in other Turkic-related languages, such as Karakalpak, Kyrgyz, and Kazakh, for cross-lingual transfer learning and the building of NLP tools with a multiplicity of linguistic support.

## 2 Background

The Uzbek language^1^ is one of the most spoken languages in the Turkic language family, and is characterized by its rich and complex agglutinative morphology [1]. Despite the rapid development of resources and models recently, the language remains relatively under-resourced in the field of NLP. Developing robust NLP tools for fundamental tasks like part-of-speech (POS) tagging, morphological analysis, and syntactic parsing requires large-scale, high-quality annotated datasets that reflect the linguistic characteristics of the language [2]. Previous efforts in Uzbek POS tagging, which are only a few, have often relied on rule-based systems or smaller datasets with limited POS tags [3,4].

Beyond part-of-speech tagging, the recent trends for Uzbek NLP have seen rapid growth through the development of diverse linguistic resources and tools, including the implementation of algorithms for Named Entity Recognition (NER) [5] and the creation of corpora for aspect-based sentiment analysis [6]. Also, the development of specialized tools for punctuation analysis [7], lemmatization and stemming [8,9] reflects a concerted effort to address the unique morphological challenges of the Uzbek language.

### 2.1 POS tagset creation

For the development of UzbekPOS, we adopted the Universal POS (UPOS) guidelines^2^ from the Universal Dependencies (UD) project^3^ to ensure the dataset's compatibility with global linguistic standards [10]. In order to capture the specific morphological and syntactic features of Uzbek at an expert level, the standard UPOS tagset was slightly adapted. The major change being the Determiner (DET) tag was intentionally omitted because Uzbek lacks articles, which form the bulk of this category in many other languages. Also, one more tag, Modal (MOD), which is not included in the UPOS tags list, was incorporated as a language-specific tag (XPOS) to reflect the unique functional structure in Uzbek grammar [11]. The final tagset list, together with their categories as well as types are presented in Table 1.

**Table 1:** The list of tags used for annotating the Uzbek raw text data, followed by their categories, types, and names.

Category	Tag	Name	Type
Open class words	ADJ	Adjective	UPOS
	ADV	Adverb	UPOS
	INTJ	Interjection	UPOS
	NOUN	Noun	UPOS
	PROPN	Proper noun	UPOS
	VERB	Verb	UPOS
Closed class words	ADP	Adposition	UPOS
	AUX	Auxiliary verb	UPOS
	CCONJ	Coordinating conjunction	UPOS
	DET	Determiner	UPOS
	NUM	Numeral	UPOS
	PART	Particle	UPOS
	PRON	Pronoun	UPOS
	SCONJ	Subordinating conjunction	UPOS
Other	PUNCT	Punctuation	UPOS
	SYM	Symbol	UPOS
	X	Other	UPOS
Language specific	MOD	Modal	XPOS

The Determiner class tag “DET” from the closed class words has been omitted in the case of the Uzbek language, since it is mostly used to tag articles and Uzbek does not have articles [12]. By aligning with the UD framework as well as considering the linguistic characteristics of Uzbek grammar, this dataset represents a valuable resource that is both standardized and linguistically authentic, which is important for training accurate Uzbek NLP applications.

## 3 Data Description

The UzbekPOS dataset is provided in a single repository, with the same annotated texts over four distinct file formats to ensure accessibility for researchers and developers across various domains. Each format is designed to serve specific user needs:

1. ***Raw annotated format file (uzpos_raw.txt)***: This version provides the raw data in a human-readable string format with <token>/<tag> structure. It is ideal for quick inspection and simple pattern-matching tasks.
2. ***TSV format file(uzpos.tsv)***: A structured, tab-separated format for easy manipulation using both spreadsheet software and basic data processing libraries. This format file is useful for those who prefer a tabular view of token-level annotations.
3. ***JSONL format (uzpos.jsonl)***: This format stores each sentence as a JSON object on a new line, facilitating integration with the latest programming environments, proving efficient for large-scale data parsing in Python, Javascript or TypeScript.
4. ***CoNLL-U format (uspos.conllu)***: Following the global standards of the Universal Dependencies project [1]. It ensures that the UzbekPOS dataset can be directly utilized by existing NLP toolkits and facilitates cross-linguistic comparative studies.

An example snippet of these formats for a single pos-annotated Uzbek sentence is presented in Table 2 below:

**Table 2:** Snippets of an example sentence from the dataset in four formats used.

Raw annotated format (.txt) sentence example	*Bu/PRON tizimda/NOUN modda/NOUN va/CCONJ energiya/NOUN almashinuvi/VERB kuzatib+kelinmoqda/VERB ./PUNCT*
*TSV format* (.tsv) sentence example	*sent_id token_id form upos* *22 1 Bu PRON* *22 2 tizimda NOUN* *22 3 modda NOUN* *22 4 va CCONJ* *22 5 energiya NOUN* *22 6 almashinuvi VERB* *22 7 kuzatib+kelinmoqda VERB* *22 8 . PUNCT*
JSONL format (.jsonl) sentence example	*{"sent_id": 22, ``tokens'': [``Bu'', ``tizimda'', ``modda'', ``va'', ``energiya'', ``almashinuvi'', ``kuzatib+kelinmoqda'', ``.''], ``upos'': [``PRON'', ``NOUN'', ``NOUN'', ``CCONJ'', ``NOUN'', ``VERB'', ``VERB'', ``PUNCT'']}*
CONLL-U format (.conllu) sentence example	*# sent_id = 22* *1 Bu _ PRON _ _ _ _ _ _* *2 tizimda _ NOUN _ _ _ _ _ _* *3 modda _ NOUN _ _ _ _ _ _* *4 va _ CCONJ _ _ _ _ _ _* *5 energiya _ NOUN _ _ _ _ _ _* *6 almashinuvi _ VERB _ _ _ _ _ _* *7 kuzatib+kelinmoqda _ VERB _ _ _ _ _ _* *8 . _ PUNCT _ _ _ _ _ _*

### 3.1 Corpus statistics and tag distribution

The created UzPOS dataset is a multi-domain resource covering the linguistic diversity of the Uzbek language as much as possible. To provide an overview of the dataset's scale and characteristics, a summary of basic counts and averages are given in Table 3. These statistics include metrics such as the total number of sentences, unique tokens, and average sentence length.

**Table 3:** Basic statistical information regarding the dataset with counts and averages.

Statistics type	Statistic metric	Quantity
Basic counts	Sentences (lines):	4412
	Token/TAG pairs:	53113
	Unique surface tokens:	16904
	Unique tags:	16
	Bad/unparsed items:	0 (None)
Averages	Avg tokens per sentence:	12
	Avg token length (chars, token only):	6.6
Special tokens	Multi-word expressions	3493
	Tokens containing emoji	24

The statistics above are provided for the entire dataset and lack per-tag information. Hence, a detailed breakdown of the 16 unique POS tags with their frequencies in the dataset, density, and type-token ratios is presented in Table 4, to better illustrate the morphological distribution within the annotated corpus. This analysis includes the density of each tag, the Type-Token Ratio (TTR) to measure lexical variation, and the most frequent tokens associated with each category. As shown in the following table, nouns and verbs constitute the majority of the annotated tokens, reflecting the core structural elements of the Uzbek language.

**Table 4:** All 16 POS tags with their frequencies, density percentage, and type-token ratios, followed by the top-5 example words/tokens taken from the dataset. TTR- Type-token ratio is a metric to display the lexical variation of the tag. The bigger the ratio more diverse the set of words used for the tag, lower the ratio means fewer words are repeatedly used for the tag.

Tag	Count	Density (%)	TTR*	Top occurring tokens
**NOUN**	17912	33.7%	0.46	*"Misol"(106), ``suv''(84), ``ega''(84), ``moddalar''(61)*
**VERB**	8854	16.7%	0.53	*"bo‘lgan"(100), ``bo‘lib''(61), ``deyiladi''(55), ``bo‘ladi''(48)*
**PUNCT**	7934	14.9%	0.004	*``.''(4033), ``,''(3167), ``–''(126), ``:''(123), ``?''(102)*
**ADJ**	4826	9.%	0.3	*``boshqa''(104), ``katta''(89), ``turli''(80), ``asosiy''(68)*
**PRON**	2669	5.%	0.12	*``U''(273), ``Bu''(156), ``u''(141), ``bu''(139), ``uning''(97)*
**ADP**	2061	3.9%	0.07	*"bilan"(539), “uchun”(278), “orqali”(92), “bo‘yicha”(74)*
**CCONJ**	1875	3.5%	0.03	*"va"(1419), “yoki”(133), “esa”(108), “hamda”(67)*
**ADV**	1480	2.79%	0.28	*"juda"(119), “eng”(96), “ko‘p”(80), “o‘zaro”(49)*
**NUM**	1260	2.4%	0.38	*"bir"(148), “ikki”(63), “birinchi”(37), “ikkinchi”(31), “2”(30)*
**PROPN**	1122	2.1%	0.52	*"O‘zbekiston"(35), “Toshkent”(21), “Lui”(20), “Eron”(15)*
**AUX**	987	1.9%	0.05	*"edi"(343), “ekan”(256), “mumkin”(94), “emish”(65)*
**SCONJ**	800	1.5%	0.08	*"chunki"(171), “deb”(95), “bo‘lsa”(92), “negaki”(58)*
**MOD**	425	0.8%	0.2	*"bor"(58), “kerak”(44), “yo‘q”(33), “mavjud”(32)*
**PART**	388	0.7%	0.07	*"ham"(266), “faqat”(34), “hech”(12), “xuddi”(9)*
**SYM**	320	0.6%	0.42	*"-"(82), “km”(14), “A”(10), “m”(8), “%”(8)*
**INTJ**	200	0.4%	0.7	*"Xo‘sh"(4), “E”(4), “Mah-mah”(4), “Hoy”(2), “Salom”(2)*
**Total**	**53841**	**100%**	**0.31**	***“.”(4033), “,”(3167), “va”(1419), “bilan”(539), “edi”(343)***

## 4 Experimental Design, Materials and Methods

This study significantly extends the dataset originally developed for the UzbekTagger project [9], which consisted of 1,581 sentences across 23 categories using a 12-tag scheme. We incorporated these initial sentences and augmented the corpus with over 2,800 newly collected sentences, introduced two additional text categories, and expanded the annotation schema to 16 tags. These enhancements were designed to improve domain coverage and enable more granular linguistic analysis. Crucially, the inherited subset underwent the same rigorous re-annotation and validation pipeline as the new data to integrate the expanded tagset, correct prior inconsistencies, and ensure uniform quality across the entire corpus.

### 4.1 Source material and data collection

The raw text corpus chosen for this task was designed to be as representative as possible of the official Uzbek language, so we collected data from various sources like websites, books, educational materials, and many more, with 25 diverse categories, ensuring a balance between formal academic registers and informal digital communication.

The UzbekPOS dataset was curated from three primary sources to ensure both linguistic diversity and legal compliance. Except for the “Interactions and Comments” category, all textual data were sourced from the following:

1. Educational and Literary Texts: A significant portion of the corpus was extracted from the Republican Youth E-Library^4^. This source provided access to openly available school textbooks and contemporary literature, ensuring the inclusion of formal and pedagogical Uzbek language structures.
2. Journalistic and News Media: Current events and media-style Uzbek were represented through data from Darakchi news platfrom^5^. The authors obtained a formal written letter of agreement from the publisher, granting permission to utilize their archival news text for research purposes.
3. Social Media and Synthetic Data: The specific category in the dataset “Suhbat va kommentlar” (Interactions and Comments) was developed to capture informal, colloquial, and digitally-mediated Uzbek. These data were manually collected by the authors and annotators from public social media channels or, where necessary to represent specific linguistic phenomena, synthetically generated by the expert team to maintain grammatical variety. Adding this section was necessary for the fact that it captures the colloquial and often morphologically non-standard use of the language in digital spaces [13].

All the fields of texts included in the dataset are listed in Table 5 with their relative counts.

**Table 5:** Names of the fields covered in the dataset with their sentence counts.

№	Category	Sentences	№	Category	Sentences
1	Adabiyot (Literature)	200	14	Madaniyat (Culture)	200
2	Anatomiya (Anatomy)	200	15	Matematika (Mathematics)	150
3	Biologiya (Biology)	200	16	Ona tili (Mother tongue)	200
4	Botanika (Botany)	200	17	Qishloq xo‘jaligi (Agriculture)	200
5	Din tarixi (Religion)	200	18	Siyosat (Politics)	150
6	Dunyo (World)	200	19	Sport (Sports)	200
7	Fizika (Physics)	150	20	Tarix (Hitory)	200
8	Geografiya (Geography)	200	21	Texnologiya (Technology)	150
9	Huquq (Law)	150	22	Tibbiyot (Medicine)	200
10	Informatika (Informatics)	150	23	Zoologiya (Zoology)	150
11	Iqtisodiyot (Economy)	150	24	San’at (Art)	112
12	Jamiyat (Society)	200	25	Suhbat va kommentlar (Interactions and comments)	150
13	Kimyo (Chemstry)	150			
Total:			4412		

### 4.2 Annotation pipeline

**Fig. 1:**
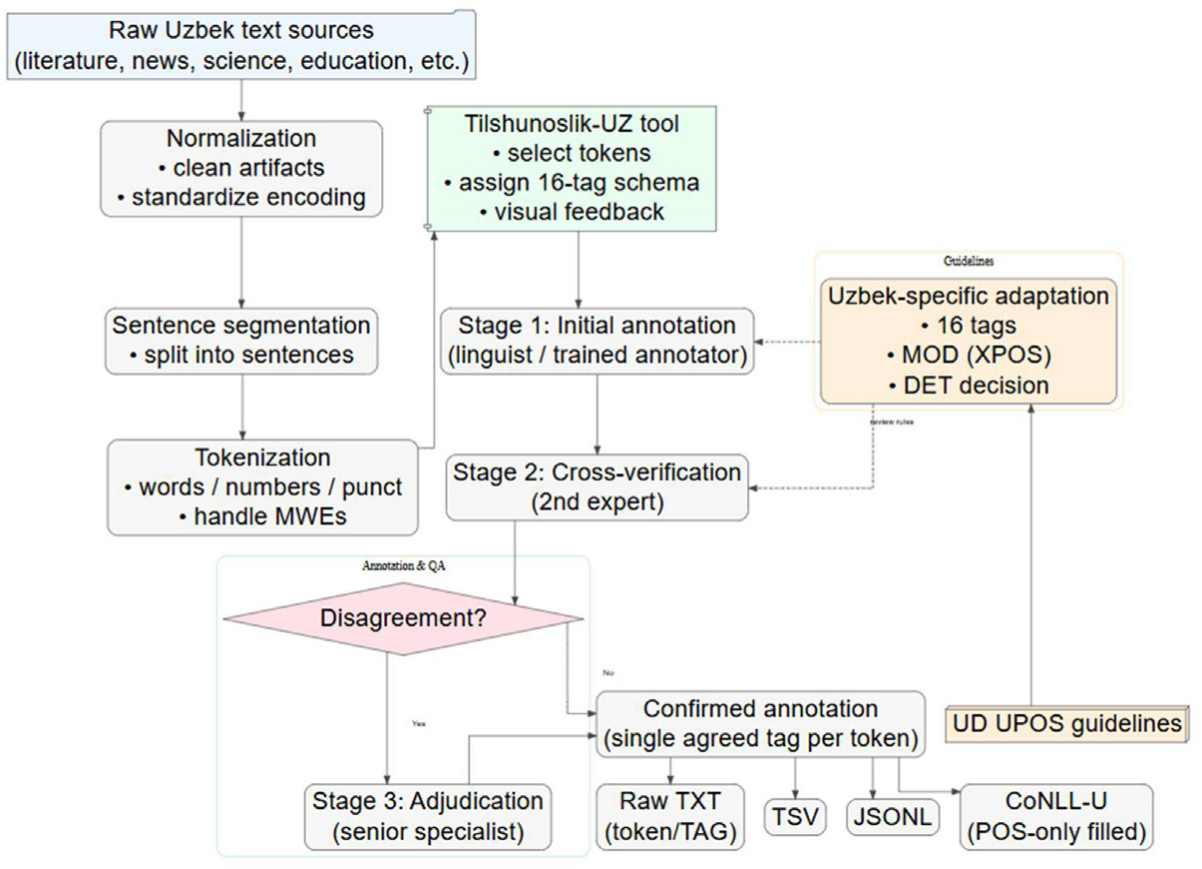
Illustration of the pos-tagging annotation process with all the stages included.

Once the quality raw text collection process is finished, an annotation pipeline was implemented that includes the steps of text normalisation, sentence segmentation, annotation, quality assurance, adjudication, as well as formatted output. The sequence and logic of all these stages are visualised in Fig. 1.

**Fig. 2:**
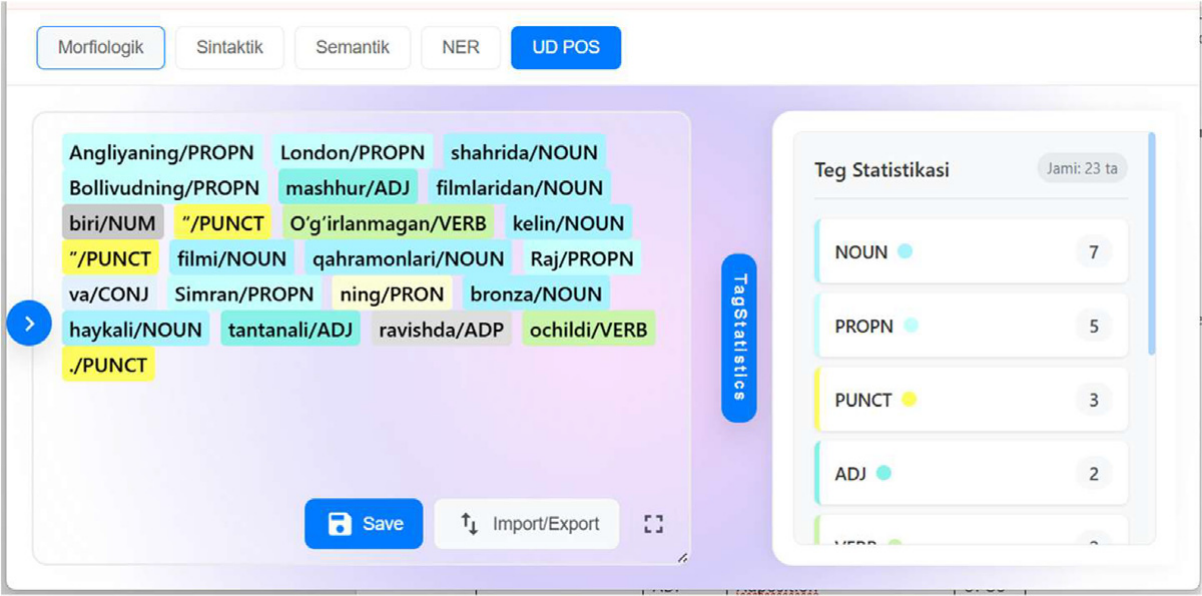
Tilshunoslik.uz POS-tagging annotation tool user interface with a sample sentence.

Although the pipeline is well-visualised, some stages require more explanation than just naming. One of those steps is the annotation tool choice, where we have used the POS-tagging annotation tool from Tilshuoslik UZ platform^6^, which is part of fundamental NLP tools for the Uzbek language created by the same authors [14]. This platform provides a user-friendly interface that allows annotators to select tokens and assign tags from the predefined 16-tag schema. The tool was designed to minimize human error by providing a streamlined workflow and immediate visual feedback for each tagged sentence. An example user interface of the annotation toolfrom the Tilshunoslik-UZ platform is presented in Fig. 2.

As for the annotation protocol and tagset adaptation, the annotation process followed the Universal Dependencies (UD) UPOS guidelines, ensuring the dataset's compatibility with international NLP standards [15]. As noted in the Background section, we adapted the standard set to fit Uzbek’s linguistic profile —specifically omitting the DET tag and utilizing the MOD (Modal) tag to capture language-specific nuances [16].

To ensure the highest possible reliability (Gold Standard), a three-stage validation process was employed:

1. ***Initial Annotation:*** Each sentence was first processed by a professional linguist or trained annotator.
2. ***Cross-Verification:*** Every annotated sentence was independently reviewed by a second expert to identify potential inconsistencies or errors.
3. ***Adjudication:*** In cases of disagreement between the first and second annotators, a senior adjudicator (a specialist in Uzbek linguistics) performed a final review to resolve the conflict and determine the correct tag.

The annotation process was conducted by a team of native Uzbek speakers to ensure linguistic precision. The team consisted of two lead experts with PhDs in AI and NLP—the first two authors of this study—and 12 Master’s students specializing in Computational Linguistics. The annotation team was assembled using a purposive convenience sampling technique, recruiting from within a specialized academic environment because accurately annotating the complex agglutinative morphology of the Uzbek language requires deep theoretical linguistic knowledge rather than relying solely on native fluency. To ensure high reliability, strict inclusion criteria mandated that all selected annotators be native Uzbek speakers holding a formal Bachelor’s degree in Uzbek Linguistics, be actively enrolled in a Master’s program for Computational Linguistics or a closely related NLP field, and successfully complete a rigorous one-month preliminary training phase focused specifically on the project's custom 16-tag schema. Conversely, the exclusion criteria strictly disqualified individuals who were non-native speakers, lacked a documented academic background in formal linguistics, or failed to demonstrate sufficient competency with the annotation guidelines during the mandatory training period prior to the commencement of the formal labelling task.

Inter-annotator agreement was measured prior to the adjudication stage using Cohen’s κ, yielding a value of 0.86, which indicates a high level of annotation consistency.

Finally, the final version of the tagged gold standard dataset was transformed into 4 different formats, from simple raw text all the way to the CONLLU format. It is worth mentioning that the dataset content and token-tag pairs are all the same in all the files reported with their own formats, except in the case of the CONLLU format, where the language-specific “MOD” tag was given the “AUX” UPOS tag in the POS tags columns, moving the original language-specific tag to the fifth column.

## Limitations

Although the dataset has large-scale, expert-annotated POS data for Uzbek, some limitations might still remain. The corpus has only part-of-speech classes, and no other kind of morphological or syntactic feature annotations were given, but this does not lower the value of the dataset. Although the whole data was annotated with multiple experts and all possible annotation conflicts were resolved at the adjudication stage, some small annotation bias could still have preserved. However, the UzbekPOS dataset has been quality-controlled and hand-curated, and it is a robust and reliable source for developing the POS tagging models for Uzbek.

## Ethics Statement

The authors confirm that the annotated dataset and additional scripts reported in this manuscript do not involve human subjects, animal testing, or any data from social networks. All the initial resources to annotate were collected from publicly available texts and some licensed materials, with special permission to obtain. We confirm that we have read and followed the publication guidelines in Data in Brief.

## Credit Author Statement

**Maksud Sharipov:** Conceptualization, Methodology, Formal analysis, Resources, Writing – Review & Editing, Supervision, Project administration; **Elmurod Kuriyozov:** Software, Investigation, Data Curation, Writing - Original Draft, Visualization; **Jernej Vičič:** Conceptualization, Investigation, Writing – Review & Editing, Validation, Funding acquisition.

## Data Availability

Mendeley DataUzbekPOS: Multi-domain Part-Of-Speech Dataset for the Uzbek Language (Original data).

## Footnotes

4 The Republican Youth E-Library with openly available books: https://kitob.uz/uz.

5 Darakchi news portal: https://darakchi.uz/oz.

## Footnotes

1 More about the Uzbek language: https://en.wikipedia.org/wiki/Uzbek_language.

2 UPOS guidelines: https://universaldependencies.org/u/pos/.

3 Universal Dependencies project: https://universaldependencies.org/.

6 Tilshunoslik UZ NLP tools platform - https://tilshunoslik.uz/.
